# An EEG study of human trust in autonomous vehicles based on graphic theoretical analysis

**DOI:** 10.3389/fninf.2022.907942

**Published:** 2022-08-16

**Authors:** Tao Xu, Andrei Dragomir, Xucheng Liu, Haojun Yin, Feng Wan, Anastasios Bezerianos, Hongtao Wang

**Affiliations:** ^1^The Faculty of Intelligent Manufacturing, Wuyi University, Jiangmen, China; ^2^The N1 Institute, National University of Singapore, Singapore, Singapore; ^3^Department of Electrical and Computer Engineering, Faculty of Science and Technology, University of Macau, Macau, Macao SAR, China; ^4^Hellenic Institute of Transport (HIT), Centre for Research and Technology Hellas (CERTH), Thessaloniki, Greece

**Keywords:** trust in automation, behavioral modeling, autonomous vehicles, brain functional network, graphic theoretical analysis

## Abstract

With the development of autonomous vehicle technology, human-centered transport research will likely shift to the interaction between humans and vehicles. This study focuses on the human trust variation in autonomous vehicles (AVs) as the technology becomes increasingly intelligent. This study uses electroencephalogram data to analyze human trust in AVs during simulated driving conditions. Two driving conditions, the semi-autonomous and the autonomous, which correspond to the two highest levels of automatic driving, are used for the simulation, accompanied by various driving and car conditions. The graph theoretical analysis (GTA) is the primary method for data analysis. In semi-autonomous driving mode, the local efficiency and cluster coefficient are lower in car-normal conditions than in car-malfunction conditions with the car approaching. This finding suggests that the human brain has a strong information processing ability while facing predictable potential hazards. However, when it comes to a traffic light with a car malfunctioning under the semi-autonomous driving mode, the characteristic path length is higher for the car malfunction manifesting a weak information processing ability while facing unpredictable potential hazards. Furthermore, in fully automatic driving conditions, participants cannot do anything and need low-level brain function to take emergency actions as lower local efficiency and small worldness for car malfunction. Our results shed light on the design of the human-machine interaction and human factor engineering on the high level of an autonomous vehicle.

## Introduction

With progressions in automotive safety technologies, both passive and active methods such as lane-centering assistance and adaptive cruise control have contributed to a considerable reduction in traffic fatalities (Van Arem et al., 2006; Lee and Litkouhi, 2012; Milanés et al., 2013). However, the driving death toll in China is still significant and human error accounts for a large proportion (Bhalla et al., 2013). Moreover, traffic congestion is another factor that contributes to traffic accidents. Hence, autonomous vehicles (AVs), which can make optimal decisions bypassing human intervention and avoiding congested routers, have attracted much interest for a long time. In addition to improving driving safety and route planning, AVs can outperform driving efficiency. Most importantly, they can create a passion for driving in people of all ages (Choi and Ji, 2015).

The society of automotive engineering (SAE) categorizes driving automation into six levels ranging from levels 0 to 5. Levels 0 and 5 represent no automation and full automation driving, respectively, in which either the driver or the vehicle independently performs all driving tasks under all conditions. Meanwhile, from levels 1 to 4, the degree of automation increases from drive assistance to high automation. The discrimination of automation levels lies in the drivers’ vigilance of the surrounding environment. Interestingly, the discrimination between levels 4 and 5 is blurred due to the condition that the vehicle performs all driving functions. Therefore, it will be interesting to investigate the impact of malfunction of the vehicle under these two degrees of automation as there will likely be a psychological difference between with and without control of a vehicle while facing an emergency for an individual. Evidence shows that the driver and the copilot exhibit different attitudes during an emergency. Therefore, the psychological difference can be regarded as the trust of humans in machines and such trust is most important to developing full automation driving with the maturity of driving automation.

Along with the increasing automation of the AV area, human-computer interaction will be fully utilized in which the human trust in AV will play a crucial role. This is because the driver is permitted to do a secondary task instead of concentrating on driving along the journey (Ma and Kaber, 2005; Carsten et al., 2012; Hergeth et al., 2016; Petersen et al., 2019). Lee and See consider trust from the organizational, sociological, interpersonal, psychological, and neurological perspectives. Trust in AVs can be defined as the human attitude toward how AVs can help achieve user goals in a situation characterized by uncertainty and vulnerability (Lee and See, 2004). They consider how the context, automation characteristics, and cognitive processes affected the appropriateness of trust. Previous studies on human trust in AV limit subjective feelings, such as a well-designed questionnaire for self-reported measurement so that it can be used to repeatedly measure the subjects’ trust in the autonomous driving process (Kraus et al., 2020). Moreover, an objective assessment was confined to testing reaction time while facing an emergency (Payre et al., 2016). Then a more comprehensive method includes heart rate measurement and the grasp of eye gaze while executing commands of the driving assistant system (Petersen et al., 2019). However, such measurements can be regarded as the achievement of the delayed and filtered signal from the brain. Therefore, Seet M. et al. (2020) utilized EEG analysis, a fast and highly correlated electrophysiology measurement for trust in AVs. Nonetheless, the analysis focused more on the power spectral density and the functional connectivity graph metrics, which lacks the analysis according to scene switching. In addition, EEG analysis was also used for the trust testing scenario in which the participants in a matrix game included both human and machine counterparts (Dong et al., 2015). Two strategies (collaboration and egoism) were used. Results demonstrated that human-like cues affected neural responses related to the partner’s capability. In contrast, in the egoism session, the trust level of predictive partners was reflected by a statistically significant capability effect in the midline electrodes. However, the EEG analysis was confined to the ERP amplitude of different nodes, which lacks the consideration of connectivity between nodes. The results described above suggested that the discrimination of EEG signals in human-computer interaction can be a potential candidate for the study of human trust in AV whereas a more comprehensive method that considers the brain’s global or local effect should be proposed.

Recently, graph theoretical analysis (GTA) for functional connectivity networks has attracted much attention. In neuroscience, because of the intricate connections inside the brain, GTA can build a network model that contains regions of interest (nodes) and their connection (edges) to represent characteristics of the brain during different tasks. In this way, both global and local effects of the brain can be analyzed for different tasks. For example, GTA can be used for the diagnosis of degenerative disease and the analysis of working memory tasks (Langer et al., 2013; Sun et al., 2014). The advantage of GTA lies in the analysis of EEG signals in a subdivision frequency (Dai et al., 2017). Many studies have shown that the amplitude of alpha activity is negatively correlated to the number of cortical resources in performing cognitive tasks (Gevins et al., 2012; Roux et al., 2012; León-Domínguez et al., 2015). Therefore, by GTA, the human brain can be modeled as a complex network and have a small-world structure at the level of anatomical as well as functional connectivity (Stam and Reijneveld, 2007).

In this study, we will adopt GTA for the analysis of human trust in AVs. First, we introduce the simulated platform and the well-designed experimental protocol in the method section. Then we show the results of behavior performance and the analysis based on graph theory. Finally, we discuss our results and provide a conclusion.

## Materials and methods

### Participants

Fifty healthy students aged from 21 to 35 (mean:23.6 *SD* = 3.06) are recruited for this study. They should have normal vision or corrected-to-normal vision with no history of any mental diseases. They are also forbidden to take any medications during the participation of this study. In addition, they should have sufficient driving experience and be aware of the basic traffic rules and norms. This study was approved by the Institutional Review Board (IRB) at the National University of Singapore. Written informed consent was obtained from participants before the study and monetary compensation was given for their participation.

### Driving platform

The driving platform consists of three 65-inch LCD screens for monitoring the driving scene, a driving console (Logitech G27 Racing Wheel; with a steering wheel, a pedalboard, and a gear shifter unit) for operating, and a host computer for the control of the simulation. More details about the experimental senior could be found in our previous studies (Wang et al., 2020a,b). Before the experiment, it takes about 2 min for the participant to get familiar with the platform, which guarantees the comfort of driving for individuals and well follow road rules and navigational instructions. The participant follows instructions of the platform in terms of hearing and vision assembling one abides by the road navigation during driving.

### Experimental protocol

Before starting and after finishing the experiment, participants are asked to fill out two trust questionnaires that help to analyze the initial feeling of the AV and the impact of the experimental procedure on trust, respectively (Jian et al., 2000).

The car drives in an urban area with a scene of cars and pedestrians on the road. There are two basic traffic scenarios that the driver should handle. One scenario is the traffic light (TL) in which the participant should stop the car before a junction while seeing the traffic light is red. The other scenario is a car approaching (CA) in which the participant is asked to stop the car before a junction without any traffic light to avoid collision with other cars. A trial of driving is defined as an encounter with a version of the aforementioned scenario. The intertrial interval is set to be about 1 km distance. The whole experiment consists of two stages one is the practice and the other is the driving simulation. In the practice stage, the driver is navigated to drive the car manually on a road without any junction. After 2-min of driving, the driving platform guides the driver to switch to autonomous mode by pressing a switching button. Then the car will run automatically without any possible crashes and malfunctions for another 2 min.

**FIGURE 1:**
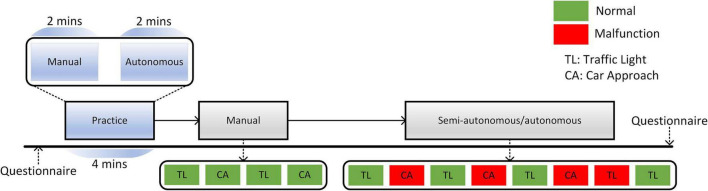
The experimental protocol of the work.

After the practice stage, it comes to the driving simulation stage, which consists of three modes: manual driving mode (SAE Level 0), semi-autonomous driving mode (SAE Level 4), and fully autonomous driving mode (SAE Level 5). In the manual driving mode, as is shown in Figure 1, participants face four junction trials, alternating between the TL and CA junctions. In the semi-autonomous mode, the trial starts with an autonomous mode which can be taken over by pressing the manual button on the steering wheel. The driver goes through eight trials arranged by the program. However, in a trial of the autonomous phase, the car condition may turn from normal to malfunction without advance notice. The malfunction is defined as the car running at the stoplight or the car still running while another car approaches the intersection. There are four trials with malfunction for both semi-autonomous driving mode and autonomous driving mode. The order of malfunction trials should fulfill the following criteria:

1. No strict trial-type alternations (or other discernable patterns) can set up the expectancy of malfunctions and unduly influence trust dynamics as the experiment progresses.
2. No more than two consecutive trials which have the same car condition (normal or malfunction). This was to avoid excessive cumulative loss of trust that would happen if there were massing of malfunctions in succession.
3. To avoid trust loss as the participant gets used to the new phase, the trial sequence must always begin with a normal trial.

At the end of each trial, the driver should guarantee the driving mode is retrieved to the autonomous driving mode. In the fully autonomous mode, the driver will repeat the drive through the routine as in the semi-autonomous mode.

### Signal processing

**FIGURE 2:**
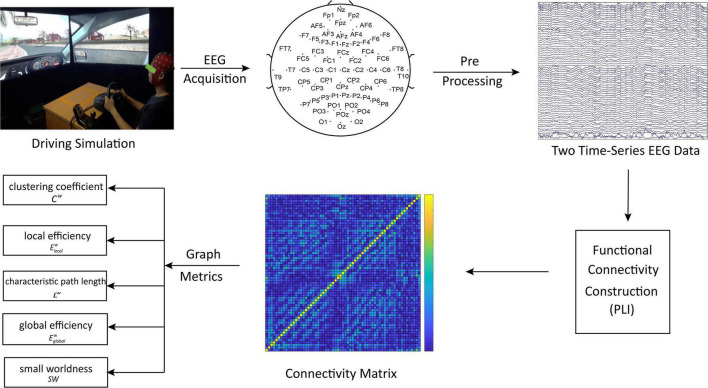
Flow chart of functional connectivity graph metrics extracted after analysis of the EEG data.

The flow chart of functional connectivity graph metrics was extracted after analysis of the EEG data as shown in Figure 2, which mainly includes parts which are EEG apparatus, EEG data pre-processing, brain functional network, and GTA.

#### EEG apparatus

During the simulated driving, EEG data are recorded using Waveguard™ caps (CA-142; ANT Neuro, Netherlands) with a sampling rate of 512 Hz and 64 Ag/AgCl electrodes whose impedance is below 15 kΩ. We also use additional electrodes to record horizontal and vertical electrooculograms (hEOG and vEOG) on both temples, as well as below and above the right eye. In particular, participants are asked to reduce unnecessary movements for the reduction of artifacts during driving.

#### EEG data preprocessing

**FIGURE 3:**
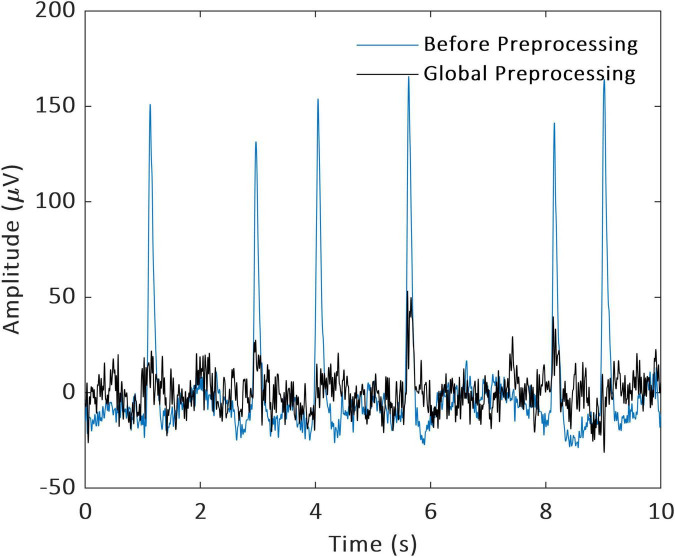
Data preprocessing.

The recorded EEG signals will be resampled to 256 Hz and bandpass filtered between 0.3 and 40 Hz. Then the processed data will be re-referenced to the left and right mastoids. At the same time, we will remove ocular and muscle artifacts with automatic artifact rejection (AAR) (Sun et al., 2014). The channels that have poor contact with the scalp will be replaced with interpolated signals of neighboring channels. Then we will do data segmentation according to the trial that the driver simulated. For each trial, 2 s were selected from the onset of the traffic light turning yellow at junctions or when the first car can be seen at intersections. In semi-autonomous or fully autonomous mode, the car may turn to malfunction during such period and thus permits us to probe into drivers’ cognitive states when they react to the AV. Finally, we will use independent component analysis to filter data with only EEG signals remaining (Figure 3).

#### Brain functional network

After the EEG data preprocessing, we employed phase synchronization (PS) to compute the statistical coupling for the functional connectivity construction in each frequency band. It is the same as the feature of EEG data, the PS was computed between two-time series. We employed the phase lag index (PLI) to estimate the degree of pairwise coupling. The EEG signals were divided by dividing the floating time window over the step for each band.

We employed the Hilbert transform to compute the instantaneous phase *z*_*i*_(*t*):


(1) $$z_{i}\,\left(t\right)=s_{i}\,\left(t\right)+j\,H\,T\,\left(s_{i}\,\left(t\right)\right)$$

where *HT*(*s*_*i*_(*t*)) is the Hilbert transform of each time series *s*_*i*_(*t*), which is estimated by:


(2) $$H\,T\,\left(s_{i}\,\left(t\right)\right)=\frac{1}{\pi }\,P.V.\int_{-\infty }^{\infty }\frac{s_{i}\,\left(t\right)}{t-\tau }\,d\tau$$

In Eq.2, *P.V.* represents Cauchy principal value. Once the phase of each time series is computed, the relative phase locking can be estimated as:


(3) $$\Delta \,\varphi \,\left(t\right)=a\,r\,g\,\left(\frac{z_{1}\,\left(t\right)\,z_{2}^{*}\,\left(t\right)}{\left|z_{1}\,\left(t\right)\right|\,\left|z_{2}\,\left(t\right)\right|}\right)$$

The PLI value ranges between 0 and 1 and is calculated with the following equation:


(4) P⁢L⁢I=|⟨s⁢i⁢g⁢n⁢φ⁢(t)⟩|

The PLI value is defined as [0,1] with 0 representing the case where there is no phase synchronization (PS), while 1 represents the perfect phase locking between two-time series.

### Graph theoretical analysis

To delve into the unknown information in the EEG data, we employed the method of GTA after building the functional connectivity network.

There were N * N adjacency matrices (*N* = 64 in this study) computed after building the functional connectivity network, which represents the connectivity structures of brain nodes. Because the functional connectivity network contains complex information and numerous useless combinations, the sparsity ranged from 10 to 20% with the step of 1% utilized in these networks, which is the ratio of the present connection number to preserve a real functional connection. In this way, we will transform the PLI matrix into a sparse matrix with different thresholds. The threshold is a proportion of the most important elements within the PLI matrix. We need to do such transformation 11 times with the proportion ranging from 10 to 20%. Hence, the weighted adjacency matrices were computed, which preserved the connection strength of the real connections. For the graph-theoretical properties with the considered sparsity, the area under the curve of the corresponding properties was extracted as features for further study.

To quantitatively investigate the topological properties of functional connectivity between the CA condition and TL condition, we implemented GTA with the Brain Connectivity Toolbox. We characterized the graph in the aspects of local segregation including clustering coefficient (*C^w^*) and local efficiency $\left(E_{l\,o\,c\,a\,l}^{w}\right)$ and global integration including characteristic path length (*L^w^*) and global efficiency $\left(E_{\mathrm{global}}^{w}\right)$ and small worldness (*SW*) based on the weighted adjacency matrices.

*C^w^* is the main indicator of information differentiation in complex network computing, which can measure the degree of aggregation within the brain functional network and reflect the possibility of each node being a neighbor. It is given by Equation (5):


(5) $$C^{w}=\frac{1}{n}\,\sum_{i\in N}\frac{2\,t_{i}^{w}}{k_{i}\,\left(k_{i}-1\right)}$$

where $t_{i}^{w}$ is the number of connections, which is the weighted geometric mean of a triangle in the neighbored node *i* and *k*_*i*_ is the number of connected nodes of *i*. *C^w^* reflects that the network forms the tendency of the local loop, and the bigger the *C^w^*. the more nodes connecting with *i*. *L^w^* is the mean of the shortest path length and is the path with the maximum total weight between vertices. It is given by Equation (6):


(6) $$L^{w}=\frac{1}{n}\,\sum_{i\in N}\frac{\sum_{j\in N,j\neq i}d_{i\,j}^{w}}{n-1}$$

where $d_{\mathrm{ij}}^{w}$ is the shortest path length between node *i* and node *j*. *L^w^* is the main indicator of global integration. The shorter the path length, the stronger the functional integration and the more direct connections between brain regions. The calculation method of *SW* is shown in Equation (7):


(7) $$S\,W=\frac{C^{w}/C_{r\,a\,n\,d}^{w}}{L^{w}/L_{r\,a\,n\,d}^{w}}$$

where $L_{\mathrm{rand}}^{w}$ and $C_{\mathrm{rand}}^{w}$ is the mean of a random network of *C^w^* and *L^w^* after 100 times random circulation. They have the same degree, node, and edge with a functional connection network. $E_{\mathrm{global}}^{w}$ measures the capability of global information transmission and is the inverse of the length of the shortest path. It is shown in Equation (8):


(8) $$E_{g\,l\,o\,b\,a\,l}^{w}=\frac{1}{n}\,\sum_{i\in N}\frac{\sum_{j\in N,j\neq i}{\left(d_{i\,j}^{w}\right)}^{-1}}{n-1}$$

$E_{\mathrm{global}}^{w}$ is a measure that evaluates the efficiency of information transfer within a region of the network. It is shown in Equation (9):


(9) $$E_{l\,o\,c\,a\,l}^{w}=\frac{1}{2}\,\sum_{i\in N}\frac{\sum_{j,h\in N,j\neq i}{\left(w_{i\,j}\,w_{i\,h}\,{\left[d_{j\,h}^{w}\,\left(N_{i}\right)\right]}^{-1}\right)}^{1/3}}{k_{i}\,\left(k_{i}-1\right)}$$

where *w*_*ij*_ is the connecting weight between node *i* and node *j*.

## Results

### Behavioral performance

The quality of the recorded data was validated by the trial-by-trial trust rating, takeover decision-making, and user preference. From the trial-by-trial trust rating, there was no difference between normal and malfunctioning trials in the semi-autonomous driving mode (*p* = 0.82). Furthermore, participants showed lower trust after car malfunction than in car normal conditions (*p* < 0.001). we also find that participants in the semi-autonomous driving mode have a stronger willingness to take over the control of the vehicle during the malfunction trials (*p* < 0.001). Finally, participants prefer the semi-autonomous driving mode to the fully autonomous driving mode (*p* < 0.001).

### Graph theoretical analysis in semi-autonomous driving mode

**FIGURE 4:**
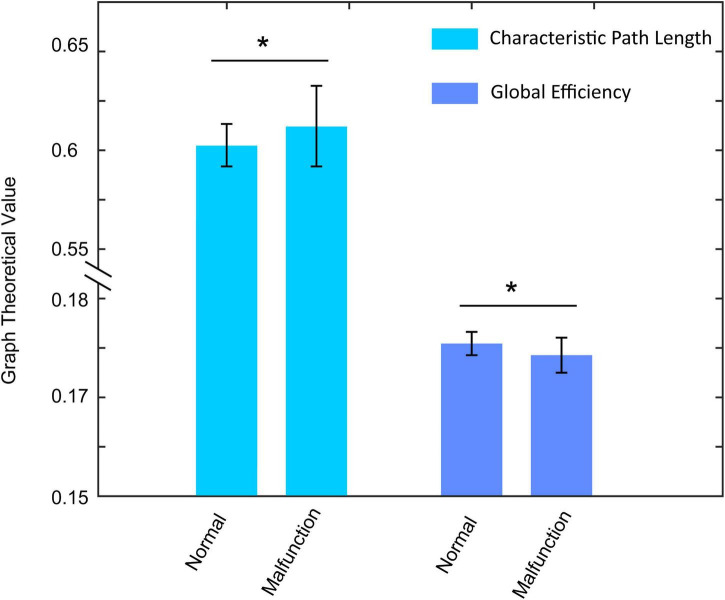
The local efficiency and clustering coefficient alterations between normal condition and malfunction condition in the CA condition. Both two indexes show significantly higher values in malfunction conditions. *p < 0.01.

The graph’s theoretical properties show significant local segregation of the brain function during the semi-autonomous driving condition. In the theta band and the car approaching condition, the participants show significant higher local efficiency [*F*_(__1_, _74)_ = 4.848, *p* = 0.031, η^2^ = 0.061, 0.192 ± 0.007 vs. 0.189 ± 0.008] and clustering coefficient [*F*_(1_, _74)_ = 6.716, *p* = 0.012, η^2^ = 0.083, 0.128 ± 0.009 vs. 0.123 ± 0.009] in malfunction trials than normal trials (Figure 4), which suggests the human brain showed more efficient information processing ability when participants encounter malfunction of a vehicle.

**FIGURE 5:**
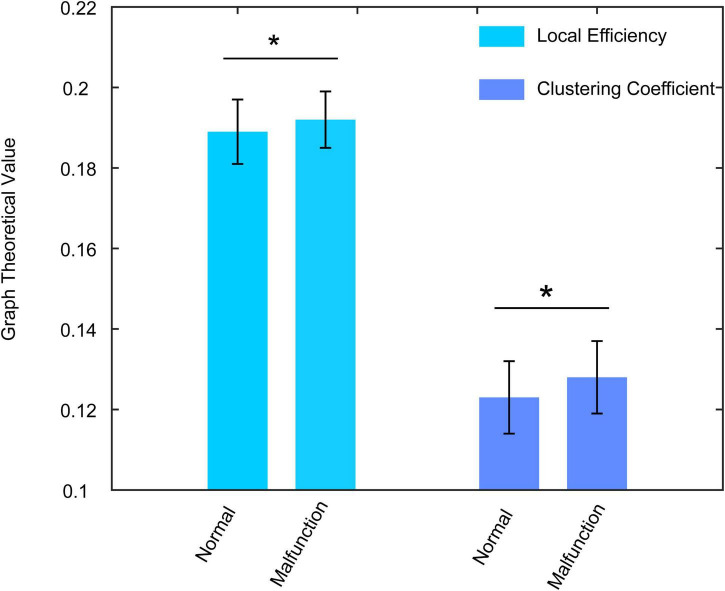
The characteristic path length and global efficiency alterations between normal condition and malfunction condition in the TL condition. Both two indexes show significantly higher values in malfunction conditions. *p < 0.01.

On the contrary, in the TL condition, participants show significant higher characteristic path length [*F*_(1_, _74)_ = 6.084, *p* = 0.015, η^2^ = 0.077, 0.608 ± 0.019 vs. 0.599 ± 0.010] and lower global efficiency [*F*_(1_, _74)_ = 6.379, *p* = 0.014, η^2^ = 0.079, 0.174 ± 0.003 vs. 0.176 ± 0.002] in the theta band during the malfunction condition (Figure 5). Such results suggest human brains have low level of information processing ability when approaching the traffic light.

### Graph theoretical properties in full automation condition

**FIGURE 6:**
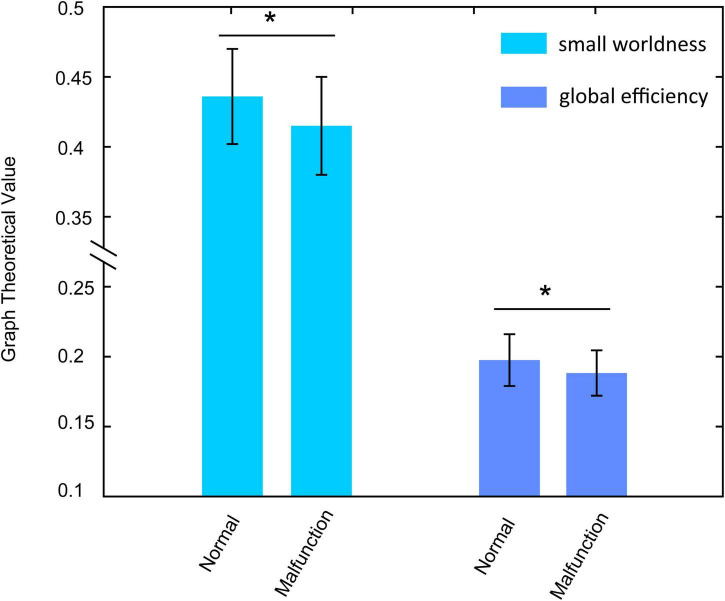
The small worldness and local efficiency alterations between normal condition and malfunction condition in the CA condition. Both two indexes show significantly lower values in malfunction conditions. *p < 0.01.

In this paper, the graph-theoretical properties in autonomous driving conditions were evaluated. In CA condition, participants show significant lower local efficiency [*F*_(1_, _74)_ = 4.491, *p* = 0.029, η^2^ = 0.063 0.188 ± 0.007 vs. 0.192 ± 0.008] in beta band during malfunction occurred (Figure 6). Furthermore, the significant lower small worldness [*F*_(1_, _74)_ = 6.982, *p* = 0.010, η^2^ = 0.086 0.415 ± 0.035 vs. 0.436 ± 0.034] was observed which means the brain has lower information processing ability in such condition (Figure 6). Such results may suggest participants cannot do anything in the fully autonomous driving condition and need low-level brain function to take emergency actions.

## Discussion

### Resource identification initiative

The extent to trust in new technology always decides the speed of the development of the corresponding technology (Gefen and Straub, 2000; Gefen et al., 2003), especially in various automation that trust is a decisive factor in the acceptance of automation (Lee and Moray, 1992; Pavlou, 2003; Lee and See, 2004; Carter and Bélanger, 2005; Parasuraman et al., 2008). For example, Jong and Yong investigated the importance of trust in adopting AVs and the factors that promote people to trust AVs (Choi and Ji, 2015). In addition, digging insights into factors that build trust can encourage a better understanding of trust in a specific item (Leimeister et al., 2005). Therefore, systematically studying AV can boost us to understand ourselves more clearly. For example, Seet M.S. et al. (2020) optimize driver-vehicle trust management according to the subtypes of trust in AV. The trust can be subdivided into competence-based trust (CT) and integrity-based trust (IT) which refer to the functional capacity of AV and the integrity of AV that will not cause deliberate harm, respectively. However, most of the analysis of trust is based on the analysis of the collected questionnaire, which is more subjective and lacks a more comprehensive consideration. These questionnaires often assume some scenarios and preconditions. However, if we encounter the scene in the questionnaire, people are often at a loss in practice. Meanwhile, survey-based investigations always give out similar conclusions in which the circumstances, as well as the performance of a robot, directly affect trust, satisfaction, and frustration. It is hard to objectively elucidate how these factors influence our trust in the interaction of human-robot let alone emergent human-vehicle interaction (Castelfranchi and Falcone, 2000; Murphy et al., 2004; Hancock et al., 2011; Abd et al., 2017). Therefore, a more objective method that seeks factors that contribute to human trust in AV is needed.

In this study, we adopt the GTA for the investigation of human trust in a high level of autonomous driving, including semi-autonomous driving (SAE level 4) and autonomous driving (SAE level 5) in a simulated driving environment. The graph-theoretical properties are efficient approaches to evaluating the function of the human brain. Particularly, the human brain showed local segregation and global integration of brain functions (Dai et al., 2017). On one hand, the local efficiency and the clustering coefficient are measurements of the brain’s local information transmission ability. On the other hand, the characteristic path length and global efficiency measured the information spreading ability of the whole brain. The local and global properties are the critical indexes to assess the brain states of different driving conditions and reflect the trust degree during driving a vehicle.

Seet M. et al. (2020) also paid attention to the EEG-based analysis of human trust in AV. And they also concluded that a reduction in trust during full automation malfunctions. However, they focus on the analysis of brain regional influence on the driving condition. For example, they found that there was a remarkable decrease in functional segregation in the right frontal area during the fully autonomous driving mode and such regional discrimination may be related to the momentary impairment of the ability to plan logically about specific actions. In contrast, our analysis focuses more on the influence of the driving scene on brain activity.

The development of human trust in AVs can be divided into several stages. The first one is to investigate human trust once there is a malfunction of the vehicle. In previous work, Seet M. et al. (2020) used self-reported trust ratings to demonstrate the difference in human trust in normal or malfunctioning driving conditions in both high automation mode and full automation mode. They found that there is no significant difference between normal and malfunction trials in high automation driving mode whereas there is a significant difference for that in full automation mode. And drivers are prone to take over the task in high automation mode once there is a malfunction. The second stage that we need to focus on is to elucidate how the brain reacts to the different scenarios during automatic driving. In this study, we focus on the analysis of the brain reaction to CA and TL conditions with different car conditions. As is shown in Figures 4, 5, there is an opposite way for the brain to process the malfunction in which it shows high local efficiency and low global efficiency for CA and TL, respectively. The opposite information-processing ability of the human brain during the CA condition and TL condition demonstrates that participants have different levels of trust during these two conditions. When the vehicle runs into a complex environment, such as an intersection without a traffic light, the participants show a low level of trust in the machine and can handle an emergency in time (higher information processing ability). However, while driving in a safe condition (TL condition), participants show a high level of trust in the machine and cannot take emergency action in time (lower information processing ability). The third stage may lie in the facilitation of human trust in AVs. However, the premise of many studies is that the driver should observe the vehicle’s performance and be ready to take over the task once there is an emergency. The secondary task is always regarded as the distraction of driving that deviates from the original intention of automatic driving. Situational awareness can help the driver to promote their trust in AVs (Miller et al., 2014). Petersen et al. (2019) changed the situational awareness with the variation of a verbal message to the driver and found that the high situational awareness condition can cause a significantly high level of trusting behavior. In the future, we can also add voice prompt with situational awareness into the experiment for the analysis of brain reaction to the AV.

Seet M. et al. (2020) also analyzed the AV malfunction on human trust. However, there are some discriminations between these two works. One of them is that the frequency band used for analysis is different. In our analysis, we focus on the theta band whereas they aim on the alpha band.

## Conclusion

In this study, a simulated driving platform with an EEG data collection system is used for the evaluation of human trust in AVs. The behavior performance shows that the driver has less trust during the fully automatic driving mode. We also used GTA to illustrate how the brain reacts to both semi-autonomous driving mode and fully autonomous driving mode. Our results have the potential to be adopted for the improvement of human trust in AV.

## Data availability statement

The raw data supporting the conclusions of this article will be made available by the authors, without undue reservation.

## Ethics statement

The studies involving human participants were reviewed and approved by Institutional Review Board (IRB) at National University of Singapore. Written informed consent for participation was not required for this study in accordance with the national legislation and the institutional requirements.

## Author contributions

TX, AB, and HW designed the study. XL and HY wrote the code. AD carried out the experiments and analyzed the data. TX wrote the manuscript. FW contributed to the conception of the study. HW helped draft the manuscript. All authors contributed to the article and approved the submitted version.
